# Preoperative vaping prevalence and behavior of French surgical patients: A multicentre study

**DOI:** 10.18332/tid/114068

**Published:** 2019-11-22

**Authors:** Yann Gricourt, Hamadi Ghezal, Pierre G. Claret, Lana Zoric, Arnaud Chaumeron, Mathieu Raux, Bastian Nucci, Emmanuel Lorne, Jean Y. Lefrant, Philippe Cuvillon

**Affiliations:** 1 Department of Anesthesiology and Pain Management, Hôpital Universitaire Carémeau, Nîmes, France; 2 Montpellier Cancer Institute (ICM), Montpellier, France; 3 Polyclinique Grand Sud, Nîmes, France; 4 Department of Anaesthesiology and Critical Care, Pitié-Salpêtrière University Hospital, Paris, France; 5 Centre Hospitalier de Bigorre, Tarbes, France; 6 Department of Anesthesiology and Intensive Care, Centre Hospitalier Universitaire Amiens-Picardie, Amiens, France

**Keywords:** e-cigarette, vaping, tobacco, preoperative period

## Abstract

**INTRODUCTION:**

Prevalence and tobacco-related perioperative complications have been largely reported. The impact of vaping (e-cigarette use) on the perioperative period has been rarely evaluated. The purpose of this multicentre cross-sectional survey was to assess the prevalence of vaping and behaviour of patients undergoing elective surgery.

**METHODS:**

After institutional review board committee and patient approvals, patients (aged ≥18 years) scheduled for elective non-cardiac surgery in six French hospitals were assessed preoperatively. Demographic characteristics, and information on vaping, smoking or dual-use status and consumption were reported.

**RESULTS:**

In six centres, 1712 eligible patients were approached and 1664 patients were included in this study from June 2016 to January 2017. Of these, 62 patients used e-cigarettes in the preoperative period (3%; 95% CI: 2–4), including 24 exclusive e-cigarette users (1%; 95% CI: 1–2), 38 dual-users (2%; 95% CI: 2–3) and 365 smokers (22%; 95% CI: 20–24). Vapers were older than smokers (53 vs 47 years old; p=0.01). During the preoperative period, 12 patients (1%) reported vaping the morning of surgery.

**CONCLUSIONS:**

In the preoperative period, vaping was ten-fold less prevalent than smoking. Although the clinical relevance may be weak, further research is needed to explore the real impact of vaping on patients’ outcomes and to elaborate on clinical recommendations.

## INTRODUCTION

Electronic nicotine delivery systems (ENDS), also called e-cigarettes, entered the European and United States (US) marketplaces around 2006 and have been proposed as a new tool in smoking cessation for millions of smokers^1-3^. Ten years later, ENDS are the most commonly used tobacco product among youths in the US^4,5^. In 2014, the prevalence of current use of e-cigarettes (vaping) was 2.4% to 3.2% in the US, with a great disparity in age, as vaping users are younger than conventional smokers^6-11^.

When used for smoking cessation, a recent Cochrane review pointed out that e-cigarettes may help to stop smoking without any serious side effects^12^. However, their role and effectiveness in managing smoking cessation is unclear and continues to divide the scientific community^13-16^. In 2016, the Food and Drug Administration (FDA) of the USA regulated a rule to cover all tobacco products, including ENDS. At the same time, the European Union (EU) Commission established new legislation to harmonise the quality and safety requirements of ENDS without banning them^7^. Unfortunately, published case reports of severe or even fatal respiratory complications have highlighted the dangers of inhaling aerosolised substances among persons who have reported use of e-cigarettes^14,15^. For the perioperative period, no data or recommendations on the use of e-cigarettes have been published, although the dangers of tobacco on airway irritation (bronchospasm, laryngospasm) and postoperative mortality are well known^16,17^. Concerning the preoperative period, no data about vaping the day of surgery and its potential consequences have been reported, in contrast to smoking^16^.

The primary aim of the present study was to assess the preoperative prevalence of current use of e-cigarettes. Secondary objectives were to describe the behaviour of French patients scheduled for elective non-cardiac surgery including vaping-cessation assessment and council from surgeons or anesthesiologists.

## METHODS

### Study design

We conducted a cross-sectional multicentre study in six French hospitals (three community and three university hospitals), representing six different French regions, using an anonymous survey presented to patients. In the six centres, 1712 eligible patients were approached, and 1664 patients were included from June 2016 to January 2017. Only 48 patients refused to participate.

### Participants

For 15 days in each centre, consecutive patients (aged ≥18 years) scheduled for elective non-cardiac surgery or endoscopic were eligible. For all patients, no specific smoking cessation counselling service was previously provided. Patients were seen by their surgeon or anesthesiologist for their procedure at least two days before the procedure. Health providers did the consultation and informed about vaping and smoking at their discretion. No written information, about vaping and smoking, was given to the patient after these preoperative consultations.

Non-inclusion criteria were: patients not wishing to consent, under guardianship, non-French speaking, and patients scheduled for obstetric analgesia (epidural) or cardiac surgery. Patients for whom a pre-anaesthetic visit could not be performed were not included (emergency surgery, time elapsed between their surgeon’s visit and the day of surgery was <48 h).

Potentially eligible patients were approached on the day of the procedure in the pre- anaesthesia room. Informed consent was obtained from those patients who agreed to participate. According to the French law, the present study was approved by the Institutional Review Board (IRB, 16.01.01, 2016, Chairman: T. Lavabre-Bertrand, Nimes, France) and registered on ClinicalTrials.gov (NCT03594643)^18^.

### Definitions of vaping and smoking status

Patients were defined as vapers if they used ENDS (every day or a few days), regardless of the nicotine content of the devices. Vapers were categorised by their nicotine level in ENDS rather than the Fagerström test for nicotine dependence. Smokers were patients who used cigarettes every day or some days at the time of the survey. Dual-users were patients who used both cigarettes and e-cigarettes every day or some days at the time of the survey.

### Questionnaire and measurements

The anonymous survey was presented to the patients before their anaesthesia and was covered in eight topics (Supplementary file, Appendix 1): participant and surgical characteristics (questions 1–3), and cigarette and e-cigarette consumption and use (questions 4–12). Questionnaires were collected via electronic (2 centres) or paper (4 centres) files.

### Objectives

The primary objective was the prevalence of vaping in the preoperative period. The secondary objectives regarded vaping-cessation information given previously and compliance to it.

### Statistical analysis

The hypothetical frequency of outcome factor in the population was estimated at 3% ± 1% (confidence limits). The design effect was set at 1 assuming a random sample. Thus, the sample size calculated for this hypothetical frequency was 1117 patients at a 95% confidence level (OpenEpi, version 3). Also, we decided to include a large cohort (>1500) to reduce potential bias (incomplete answer).

We examined data before analysis to ensure that the assumptions of statistical models were satisfied using Shapiro-Wilk statistics. Patient characteristics are expressed in absolute number with percentages for categorical variables, and means with standard deviation (SD) or medians with interquartile according to the distribution for continuous variables. Qualitative variables were compared by chi-squared or Fisher exact (when necessary) tests. Quantitative variables were compared by Student’s t-test or Mann-Whitney test. All statistical tests were two-sided. We considered as statistically significant a p-value <0.05. We performed analysis using R version 3.1.2 (R Foundation for Statistical Computing, Vienna, Austria).

## RESULTS

The distribution of surgical procedures and patient characteristics are shown in Table 1. No sex ratio difference was found between smokers and vapers (p=0.78), however smokers were younger than vapers (47 vs 53 years old; p=0.01).

**Table 1:** Characteristics of the patients included in the preoperative period and surgery (procedure and hospitalization), from six French Hospitals, 2016–2017

*Characteristics*	*Total*	*Vaper*	*Smoker*	*Dual user*	*Non-smoker*
N (%)	1664	24 (1)**	365 (22)	38 (2)**	1237 (74)**
(95% CI)	-	(1–2)	(20–24)	(2–3)	(72–76)
**Age** (years)					
mean (SD)	57 (20)	51 (13)**	47 (14)	48 (12)	61 (20)**
(95 % CI)	(19–22)	(12–14)	(12–15)	(12–13)	(18–21)
**Sex ratio**					
(Male/Female)	779/885	7/17	178/187	16/22	561/661
(95% CI), male	(44–49)	(40–42)	(45–50)	(40–42)	(41–44)
**Employment,** n (%)					
None	196 (12)	4 (16)	72 (19)	7 (18)	113 (9)
Student	22 (1.3)	0	7 (1)	1 (3)	12 (1)
Worker	638 (38)	11 (45)	203 (56)	21 (55)	401 (33)
Pensioner	743 (45)	5 (20)	64 (17)	5 (13)	660 (54)
Other	0	2 (8)	23 (6)	3 (8)	36 (3)
**Surgical procedure,** n (%)					
Orthopedic	402 (24)	5 (21)	101 (28)	10 (26)	282 (23)
Abdominal	232 (14)	3 (12)	57 (16)	10 (26)	170 (14)
Urologic	230 (14)	2 (8)	39 (11)	4 (10)	185 (15)
Gynecologic	188 (11)	2 (8)	46 (13)	3 (8)	138 (11)
Ophthalmologic	119 (7)	1 (4)	15 (4)	2 (5)	101 (8)
Vascular	115 (7)	3 (12)	23 (6)	4 (10)	84 (7)
Endoscopic	109 (6)	3 (12)	15 (4)	1 (3)	90 (7)
Otolaryngologic	88 (5)	2 (8)	22 (6)	1 (3)	59 (5)
Plastic	67 (4)	4 (16)	18 (5)	0	44 (4)
Other surgery	114 (6)	0	33 (9)	6 (15)	67 (5)
**Hospitalization**					
Ambulatory					
n (%)	907 (54)	14 (58)	195 (53)	19 (50)	678 (55)
(95 % CI)	(52–57)	(57–59)	(52–53)	(50–51)	(53–57)
Conventional					
n (%)	757 (46)	10 (41)	156 (43)	17 (45)	523 (43)
(95 % CI)	(43–48)	(40–42)	(42–43)	(44–46)	(42–44)

Results are: number (%=100×n/N, rounded), mean (SD), and CI (confidence interval).

* p<0.05 compared to smoking.

** p<0.01 compared to smoking.

In the studied population, 62 patients used e-cigarettes in the preoperative period, 3% (95% CI: 2–4), including 24 exclusive e-cigarette users 1% (95% CI: 1–2) and 38 dual-users (2%; 95% CI: 2–3) (Table 1). Vapers reported that before they started e-cigarette use, they smoked on average for 25 (22) years (95% CI: 24–27) and used ENDS devices for 2 (1) years (95% CI: 1–2), with daily use in 90 % of cases. The reported nicotine level (mg/mL) used was: 0–6 for 20 patients, 7–12 for 13 patients and >12 for 15 patients, and unknown for 14 patients. In the six centres, no information was provided to the patients for vaping cessation. During the preoperative assessment, 12 of 1664 patients (1%) reported vaping on the morning of surgery (Table 2).

**Table 2:** Use of cigarette (smoking), e-cigarette (vaping) or dual use, the day before or day of surgery and instructions to the patients (at least 2 days before surgery)

	*All*	*Vaping*	*Smoking*	*Dualuser*
N	387	24	331	32
**Patients informed about risk for preoperative period**				
About smoking				
n (%)	145 (37)	-	134 (40)	11 (34)
(95% CI)	(35–38)	-	(39–41)	(33–35)
About vaping				
n (%)	3 (1)	3 (14)**	-	0
(95% CI)	(0–1)	(13–14)	-	(0–1)
**Patients informed about smoking-cessation program**				
n (%)	62 (16)	-	56 (17)	6 (18)
(95% CI)	(15–17)	-	(16–18)	(17–20)
**Patients informed to stop smoking and vaping before surgery**				
For smoking				
n (%)	139 (35)	-	127 (38)	12 (37)
(95% CI)	(33–37)	-	(36–39)	(36–38)
For vaping				
n (%)	8 (0)	7 (30)**	-	1 (3)
(95% CI)	(0–1)	(29–31)	-	(2–3)
**Patients succeed for smoking-cessation before surgery**				
n (%)	68 (17)	-	65 (19)	3 (9)*
(95% CI)	(16–17)	-	(18–20)	(9–10)
Days before surgery				
n (%)	5 (0)	-	5 (0)	6 (22)
(95% CI)	(0–1)	-	(0–1)	(21–23)
**Patients vaping**				
Day before surgery				
n (%)	29 (7)	16 (66)**	NA	13 (40)**
(95% CI)	(6–7)	(63–68)	-	(39–42)
Morning of the surgery				
n (%)	12 (0)	7 (17)**	NA	5 (28)**
(95% CI)	(0–1)	(16–18)	-	(27–30)
**Patients smoking**				
Day before surgery				
n (%)	233 (60)	NA	213 (64)	20 (62)
(95% CI)	(60–63)		(62–65)	(61–63)
Morning of the surgery				
n (%)	29 (0.7)	NA	26 (8)	3 (9)
(95% CI)	(0–1)	-	(7–8)	(8–10)

Results are: number (%=100×n/N, rounded), CI (confidence interval).

* p<0.05 compared to smoking.

** p<0.01 compared to all.

Of the 1664 patients, 22% (95% CI: 20–24) of patients were smokers. Details about cigarette consumption, smoking-cessation information and preoperative use are shown in Table 2. In the studied population, 1237 (74%) did not smoke and 510 (31%) had quit smoking in the past 15 (24) years (95% CI: 15–20).

## DISCUSSION

The present multicentre cross-sectional study is the first to report the use of e-cigarettes (3%) in the preoperative period in France. Also, this study highlighted the absence of preoperative recommendations that could have been given to the patients.

The prevalence of vaping in the present study was 3%, which is within the range reported in the European or US ‘non-surgical’ population^7-9^. In a large European population survey, vaping was reported in 2.3% of cases (95% CI: 2.1–2.6)^8^. In a similar demographic survey conducted in the US, between 2013 and 2014, the prevalence of vaping was 2.6% over the previous five days^9^. Three years later (2016), the US prevalence has not changed and has been estimated at 3.2% for adults and 11.3% for high school students^10^.

As King et al.^3^ recently reported, age appears to be a key determinant of prevalence. Our study did not focus on the population aged <18 years, but we did show a higher prevalence among younger participants (aged <47 years). In the preoperative period, a pilot study10 conducted in 2014 analyzed the attitudes of smokers who were scheduled for elective surgery and found 21% of patients reporting vaping, far exceeding our results. However, this study was at one centre and did not represent the entire preoperative population compared to the present study.

In the present study, former smokers are significantly more frequent than those vaping, (3% vs 22%). The potential consequences of vaping could probably be small compared to the well-known impact of tobacco in the perioperative period. In addition, only 1/3 of those vaping were not exclusive users of e-cigarettes. Inhaled nicotine aerosol devices are not uniform and can take many forms that can be identified in the preoperative period. The inhaled nicotine aerosol dose was reported also in this study, but the number of patients included was limited to draw firm conclusions. In addition, we did not analyze recent types of products such as JUUL^3^.

This study showed that vaping was continued the day before the procedure and used on the day of the procedure. In addition, few or no recommendations were suggested to the patient. This point should be discussed in the future regarding the potential risks of these devices and the need for professionals to inform their patients^14-16^.

### Limitations

This multicentric study only concerned French centres and it could be argued that there is a difference between French and European vaping habits. However, the latest publications tend to show similar impacts in most European countries, except in England where vaping has been promoted more strongly^19^. Our study was a self-report of vaping and cigarette use, which can lead to data errors. The vaping definition was wide, without distinction between regular and occasional users of e-cigarettes and between the ENDS with and those without nicotine. This imprecise definition of the different vaping models could be a bias in the present study as recently reported by King et al.^3^ in their analysis of the e-cigarette market. Even with the uncertainties of an imprecise definition, this study reports a vaping prevalence of <5%. As the consequence of vaping in the preoperative period has not been clearly and obviously reported, the consequences of vaping could be questioned. A recent study highlighted potential respiratory illness related to e-cigarettes and another demonstrated high incidence of respiratory complications after major surgery^14,15^. Also, the safety of vaping in the perioperative period regarding bronchospasm and infection after major surgery under general anaesthesia could be questioned. Stopping or continuing vaping should be the subject of a large cohort study evaluating postoperative morbidity outcomes.

## CONCLUSIONS

Among 1664 patients, less than 3% were vapers and 22% were smokers. As the side effects of vaping have never been demonstrated, further research is needed to explore the real impact of vaping on patients’ outcomes and recommendations, i.e. whether vaping should stop or continue on the day of surgery.
