# Supplementary material for: Preoperative vaping prevalence and behavior of French surgical patients: A multicentre study
Source: Tob Induc Dis. 2019 Nov 22;17:84. doi: 10.18332/tid/114068 (PMC6897047; doi:10.18332/tid/114068)
Supplement: Supplementary file 1 [file TID-17-84-s1.pdf]

Supplementary file, Appendix 1

1. About you:

You are: ❑ A woman ❑ A man
Your age: I__I I__I years
Occupation:
❑ Student ❑ Actif ❑ Retired ❑Unemployed ❑ Other:

Your surgery is:

❑ Endoscopy (gastroscopy or colonoscopy) ❑ Orthopedics ❑Digestive ❑Urology❑ Gynecology ❑ ENT ❑ Ophthalmology ❑ Dentistry ❑ Obstetrics
❑ Neurosurgery ❑ Plastic ❑ Thoracic Vascular ❑ Cardiac
❑ Interventional Radiology ❑ Other (............................)

About your hospitalization:
❑ Outpatient (entered this morning)
❑ Hospitalized

2. Regarding nicotine cigarette and / or the electronic cigarette, you are:

1: ❑ No smoking (neither nicotine cigarette nor e-cigarette)
(The questionnaire is completed, thank you for your participation)

2: ❑ Former smoker (nicotine cigarette not e-cigarette).
You have stopped smoking for how many years I__ __I
(The questionnaire is completed, thank you for your participation)

3: ❑ E-cigarette and former smoker (you only consume e-cigarette)
Do you consume e-cigarette last night: ❑ yes ❑ no
Do you consume e-cigarette this morning: ❑ yes ❑ no
(Please turn the page and go to question 3)

4: ❑ Smoking without consume e-cigarette:
Have you smoked last night: ❑ yes ❑ no
Did you smoke this morning: ❑ yes❑no
(Please turn the page and go to question 4)

5: ❑ Smoking nicotine cigarette and e-cigarette (daily or occasionally)
Have you smoked nicotine cigarette last night: ❑ yes ❑ no
Have you smoked e-cigarette last night: ❑ yes ❑ no
Have you smoked nicotine cigarette this morning: ❑ yes ❑ no
Have you smoked e-cigarette this morning: ❑ yes ❑ no
(Please turn the page and go to question 3 and 4)

3. **If you use the electronic cigarette (e-cigarette)**

Before consume, how long have you smoked nicotine cigarette? I__I I__I years
How long do you use the electronic cigarette: I__I I__I years
How do you use the electronic cigarette: ❑ daily ❑ occasionally
Do you smoke nicotine cigarette while consume e-cigarette? ❑ Yes ❑ no

For the day of your surgery:
- Your surgeon or the anesthesiologist gave you instructions about your e-cigarette use?
❑ Yes ❑ no
- Does you were advised not consume e-cigarette before the operation this morning?
❑ Yes ❑ no
What electronic cigarette you use dosage:
❑ Do not know ❑6 mg ❑11/12 mg ❑16/19 mg
How to recharge or volume you use per week? I__I I__I recharge or mL

4. **If you smoke nicotine cigarette**

- How many years have you smoked? I__I I__I years
- Have you had preoperative instructions on tobacco by the surgeon or the anesthesiologist: ❑ Yes ❑ no
- You has been advised to stop smoking? ❑ Yes ❑ no
If so how many days before the operation I__I I__I days
If so, have you managed to stop smoking? ❑ Yes ❑ no (failed)
- You got your advised to follow a program stop or reduce smoking?
❑ Yes ❑ no

© 2019 Gricourt Y. et al.
